# Risk factors of prolonged ventilation after thymectomy in thymoma myasthenia gravis patients

**DOI:** 10.1186/s13019-021-01668-8

**Published:** 2021-09-27

**Authors:** Anqi Du, Xiao Li, Youzhong An, Zhancheng Gao

**Affiliations:** 1 Department of Critical Care Medicine, Peking University People’s Hospital, Beijing, People’s Republic of China; 2 Department of Thoracic Surgery, Peking University People’s Hospital, Beijing, People’s Republic of China; 3 Department of Respiratory and Critical Care Medicine, Peking University People’s Hospital, Beijing, People’s Republic of China

**Keywords:** Myasthenia gravis, Thymectomy, Ventilation, Risk factors

## Abstract

**Background:**

To explore the risk factors for prolonged ventilation after thymectomy in patients with thymoma associated with myasthenia gravis (TAMG).

**Methods:**

We reviewed the records of 112 patients with TAMG after thymectomy between January 2010 and December 2019 in Peking University People’s Hospital. Demographic, pathological, preoperative data and the Anesthesia, surgery details were assessed with multivariable logistic regression analysis to predict the risk of prolonged ventilation after thymectomy. A nomogram to predict the probability of post-thymectomy ventilation was constructed with R software. Discrimination and calibration were employed to evaluate the performance of the nomogram.

**Results:**

By multivariate analysis, male, low vital capacity (VC), Osserman classification (IIb, III, IV), total intravenous anesthesia, and long operation time were identified as the risk factors and entered into the nomogram. The nomogram showed a robust discrimination, with an area under the receiver operating characteristic curve (AUC) of 0. 835 (95% confidence interval [CI], 0.757–0.913). The calibration plot indicated that the nomogram-predicted probabilities compared very well with the actual probabilities (Hosmer–Lemeshow test: *P* = 0.921).

**Conclusion:**

The nomogram is a valuable predictive tool for prolonged ventilation after thymectomy in patients with TAMG.

**Supplementary Information:**

The online version contains supplementary material available at 10.1186/s13019-021-01668-8.

## Introduction

Myasthenia gravis (MG) is an acquired autoimmune disease characterized by the weakness of skeletal muscle induced by neuromuscular connection disorder. Thymoma, the most common thymic epithelial tumor in the anterior mediastinum, can occurs in 10–30% of MG patients. In turns, MG is detected in 20–60% of thymoma patients [1]. These constitute a real subtype of disease named thymoma associated with myasthenia gravis (TAMG) [2]. As it has been suspected that thymoma or thymus plays an important role in the pathogenesis of MG, thymectomy is the most important factor in controlling myasthenia and influencing survival in patients with thymoma, especially for TAMG patients with 40–90% symptom remission [3, 4]. However, remission of symptoms may take weeks or months to achieve remission in these patients. And what’s even worse is that for patients who did not respond to thymectomy will further deteriorate due to the the stress of surgery and anesthesia. The need for prolonged mechanical ventilation is influenced by preoperative conditions and various perioperative risk factors. Weaning is especially challenging in the myasthenic patients, with a 25–40% failure rate [5, 6]. Leventhal et al. [7] firstly proposed a preoperative scoring system to predict the need for postoperative ventilation in myasthenic patients after transsternal thymectomy, which consists of duration of MG, history of chronic respiratory diseases, the basic dose of brombistigmine ≥ 750 mg per day, and vital capacity less than 2.0 litters. In contrast, both Grant et al. [8] and Gracey et al. [9] failed to substantiate this predictive model, and though the severity of myasthenia, the history of myasthenic crisis(MC), and the presence of thymus may be more meaningful in predicting the need for post-thymectomy ventilation. Besides, Naguib et al. [10] reported that pulmonary function test result is the predominantrisk factor for postoperative prolonged ventilation in MG patients. Despite these data, it remains uneasy to predict the requirement for postoperative ventilation for MG patients as the development of surgery, anesthesia and muscle relaxants protocol [11]. In addition, all studies to date have small sample sizes and rarely analyzed the effects of surgery and anesthesia factors on prolonged mechanical ventilation.

The nomogram has been accepted as a reliable tool to create user-friendly graphical representations of complex models to generate the probability of an event based on the individual profile of each patient [12, 13]. Therefore, we retrospectively reviewed data from a tertiary teaching hospital in mainland China to summary the characteristics of patients with TAMG, to explore the preoperative and intraoperative risk factors associated with prolonged mechanical ventilation after thymectomy and toconstructed a risk assessment nomogram model.

## Materials and method

### Study design and patients

All MG patients who underwent thymectomy in the intensive care unit of Peking University People’s Hospital between January 2010 and December 2019 were retrospectively collected. Exclusion criteria were patients who met one of the following characteristics: (1) age < 18 years, (2) the postoperative pathological diagnosis conclusion was not thymoma, (3) with incomplete clinical data (lab tests, chest radiography, et al. The diagnosis of MG was confirmed through the clinical presentation, positive AChR antibodies, and/or single-fiber electromyography results [14].

All patients received computed tomography (CT) scans before the operation to evaluate the size of the thymic tumor. The medical therapy is decided based on the effect on muscle strength and dose-dependent side effects. Radical thymectomy is completed removing all soft tissue in the anterior mediastinum between the two phrenic nerves and must be performed during a stable phase of the disease [15] by video-assisted thoracoscopic surgery (VATS) or the open thoracotomy (OT)approach. General anesthesia by intravenous-inhalation or total intravenous is conducted according to a local protocol. Other anesthesia, analgesia and nondepolarizing neuromuscular blockers agents (NMBAs) are used depending on the preference of each attending anesthetist. One-lung ventilation with double-lumen tube is used in most patients. During the operation, heart rate (HR), electrocardiography, invasive blood pressure and pulse oxygen saturation are monitored continuously. Blood gas is monitored if necessary. Extubation is attempted for all patients at the end of surgery in the operating room and was performed when the patient was responsive, the muscle strength recovered to grade IV or above, and could pass spontaneous breathing test (SBT) [16]. If failed to meet any of these criteria, patients will be transferred to ICU for a progressive weaning protocol. According to the patient’s condition, the attending and respiratory therapist responsible for the patient reduced the amount of respiratory support gradually. The final decision for extubation is dependending on the result of SBT with low-level pressure support. If the patient failed in SBT, advance ventilation support will be given immediately and reevaluated the next day. Noninvasive ventilation can be used after extubation to prevent reintubation or to complete weaning in patients with persistent requirements of ventilator support. Extubation failure [16] is defined as the need for reintubation within 48 h after extubation or death during ventilation.

Patients are classified into two groups: (1) Group 1: Non-prolonged ventilation group for those who extubate in the operating room or shortly ventilation (< 24 h) after admission in ICU, and (2) Group 2: prolonged ventilation group for those patients who need prolong ventilation more than 24 h after surgery.

Data were collected for each patient, including age, sex, body mass index(BMI), coexistent illness, duration of MG, severity of MG (Osserman scale) and pulmonary function, history of MC, preoperative medical therapy for MG (anticholinergic drugs, immune agents, gamma globulin and plasma exchange), tumor size, method of surgical and anesthetic, use of sedation, analgesia and neuromuscular blocking drugs, blood loss, duration of operation, perioperative complications, and the postoperative data included methods of analgesia, the duration of mechanical ventilation was calculated as the duration from the end of the surgery to tracheal extubation, and the incidents of myasthenic or MC, pulmonary compliance.

### Statistical analysis

Collected data were recorded into a database which was subsequently analyzed with SPSS (Version 25, SPSS Inc., Armonk, NY, USA). The number (percentage) for categorical variables and mean (SD, standard deviation) or the median (p25, p75) for continuous variables was calculated after a normality test. The differences between the two groups were compared using the Mann–Whitney *U*-test for continuous variables and the χ^2^ test or Fisher’s exact test for categorical variables. All tests were two-sided and *P* < 0.05 was considered statistically significant.

All following statistical analyses were performed using R software (version 1.3.1093). Collinearity between variables was excluded prior to modeling based on the variance inflation factor (VIF); variables associated with a higher risk of prolong ventilation after hymectomy (*P* < 0.3) on univariate regression analysis were introduced into the backward multivariable logistic regression analysis. A nomogram was formulated based on the results of multivariate analysis by using the package of rms [17] in R software. The discriminatory ability of the model was quantified using the AUC. Calibration of the nomogram was assessed by plotting the observed outcome probabilities and the probabilities predicted by the logistic model. The Hosmer–Lemeshow test was used to examine how well the percentage of the observed probability matched the percentage of predicted probability over deciles of predicted risk.

## Results

### Demographic data

A total of 112 patients (62 females and 50 males; age, 54.0 ± 12.2 years) were collected. The flowchart of patient inclusion is shown in Additional file 1: Fig. S1. 74 patients in Group 1extubated within 24 h after surgery, and 38 patients in Group 2 need prolonged ventilation more than 24 h after surgery. Overall, the average ventilation duration is 36.8 (29.1, 155.1) h. 7 patients need prolonged ventilation more than 7 days. Moreover, 13 patients from Group 2 failed weaning. Disease severity of them was categorized as Osserman’s class I 34 (30.4%), IIa 29 (25.9%), IIb 37 (33%), III 10 (8.9), and IV 2 (1.8%). Group 2 had more patients with bulbar symptoms [24 (32.4) vs. 25 (65.8), *p* = 0.001]. The average duration of symptoms before surgery is 60 (30, 80) days. 55 (49.1%) patients were treated preoperatively with pyridostigmine bromide (0–480 mg/day), 11 combined with immunosuppressive medications, 2 with intravenous immune globulin, the other 67 patients revived no medication due to mild symptoms. No one suffered MC before surgery. All patients underwent extended thymectomy during a stable phase of the disease. The details of demographic data are shown in Table 1 and Additional file 2: Table A.

**Table 1:** Baseline characteristics

Parameter	Group 1 (n = 74)	Group 2 (n = 38)	*Z/χ*^2^	*P*-value
Age(years), *M (p25, p75)*	51 (45,60)	52 (40,67)	− 0.151	0.880
Male, n (%)	27 (36.5)	23 (60.5)	5.871	0.015
BMI(kg/m^2^), *M (p25, p75)*	24.2 (20.8,26.7)	24.3 (21.8,26.2)	− 0.172	0.863
Smoking, n (%)	9 (12.2)	13 (34.2)	7.733	0.005
VC(L), *M (p25, p75)*	3.18 (2.64,3.84)	3.03 (2.36,3.82)	− 1.000	0.317
Complications, n (%)				
Cardiovascular disease	6 (8.1)	2 (5.3)	0.028	0.868
Hypertension	14 (18.9)	8 (21.1)	0.072	0.788
Pulmonary disease	7 (9.5)	2 (5.3)	0.165	0.684
Osserman classification, n (%)				
I + IIa	50 (67.6)	13 (34.2)	11.352	0.001
IIb + III + IV	24 (32.4)	25 (65.8)		
Basic dose of pyridostigmine(mg/day), *M (p25, p75)*	0 (0.180)	180 (0,180)	− 1.906	0.057
Duration of the disease(day), *M (p25, p75)*	60 (30.190)	90 (30.225)	− 0.127	0.899
Size of thymomas (cm), *M (p25, p75)*	4 (3.6)	5 (3.8)	− 1.291	0.197

*BMI* Body mass index, *VC* vital capacity, *APACHII* acute physiology and chronic health evaluation

### Anesthesia, perioperative and pathological data

In our studies, extended thymectomy was performed under general anesthesia. Intravenous-inhalational anesthesia was used in 68 patients, total intravenous anesthesia was used in 44 patients, 7 patients combined with a nerve blockage and there was no difference between the two groups (*p* = 0.662). Nondepolarizing NMBAs was used in 92.9% (104/112) of the patients. Besides, 32 patients also used muscle relaxant antagonists, and there was a significant difference between the two groups [29 (39.2) vs. 3 (7.9), *p* = 0.001].

VATS thymectomy was performed in 100 patients, but four were converted to OT due to invasion of the major vessels. Transsternal thymectomy was performed in 16 patients and 11 of them were in Group 2. The surgery method was significant between the two groups, OT was associate with high a risk of prolonging ventilation [5 (6.8%) vs. 11 (28.9%), *p* = 0.001]. No surgical complications, including hemorrhage, reoperation, chylothorax or pneumothorax were observed in any patients. But patients in Group 2 had more blood loss, longer operation duration, more phrenic nerver or diaphragm injury, and more need of atrial pericardium and lung resected due to the invasion by the tumor (*p* < 0.05). The details of the anesthesia, perioperative and pathological data are listed in Table 2.

**Table 2:** Anesthesia, operation and pathological data

Parameter	Group 1 (n = 74)	Group 2 (n = 38)	*Z/χ*^2^	*P* value
Anesthesia method, n (%)				
Intravenous-inhalational anesthesia	46 (62.2)	22 (57.9)	*0.192*	*0.662*
Total intravenous anesthesia	28 (37.8)	16 (42.1)		
Nondepolarizing NMBAs	69 (93.2)	35 (92.1)	0.000	*1.000*
Muscle relaxant antagonist	29 (39.2)	3 (7.9)	12.048	0.001
Surgical procedure, n (%)				
VATS, n (%)	69 (93.2)	27 (71.1)	10.097	0.001
OT	5 (6.8)	11 (28.9)		
Phrenic never or diaphragm injury, n (%)	14 (18.9)	15 (39.5)	5.528	0.019
Vasoactive agents, n (%)	31 (41.9)	17 (44.7)	0.083	0.773
Plumonary wedge resection	7 (9.5)	9 (23.7)	4.149	0.042
Pericardiotomy	13 (17.6)	16 (42.1)	7.878	0.005
Operative blood loss(ml), *M (p25, p75)*	50 (20, 100)	100 (50, 300)	− 3.807	< 0.001
Operation time (min), *M (p25, p75)*	140 (110, 180)	200 (148, 280)	− 4.135	< 0.001

*NMBAs* Neuromuscular blockers agents, *VATS* video-assisted thoracoscopic surgery, *OT* open transsternal, *WHO* World Health Organization, *MNT* micronodular thymoma with lymphoid

### Univariable and multivariable analyses risk factors for prolong ventilation after thymectomy in TAMG patients

The results of the univariable analysis by R software is listed in Additional file 3: Table B. And then variables associated with a higher risk of prolonged ventilation (*P* < 0.2) on univariate regression analysis were introduced into the multivariate model. Multivariable analyses demonstrated that female, lower VC, Osserman classification above IIb, total intravenous anesthesia, and operation time above 180 min are independent risk factors, shown in Table 3.

**Table 3:** Multivariable analyses for the risk factors of extubation failure

Variables	VIF	OR(95%CI)	*P* value
Sex (male)	1.676	0.147 (0.028, 0.475)	0.002
VC	1.495	0.534 (0.261, 0.997)	0.043
Osserman classification (IIb, III, IV)	1.167	5.940 (2.192, 18.062)	< 0.001
Anesthesia method (total-intravenous)	1.156	2.295 (1.220, 6.851)	0.020
Operation time (> 180 min)	1.172	9.777 (3.355, 32.692)	< 0.001

*VC* Vital capacity, *VIF* variance inflation factor

### Prediction nomogram

**Fig. 1:**
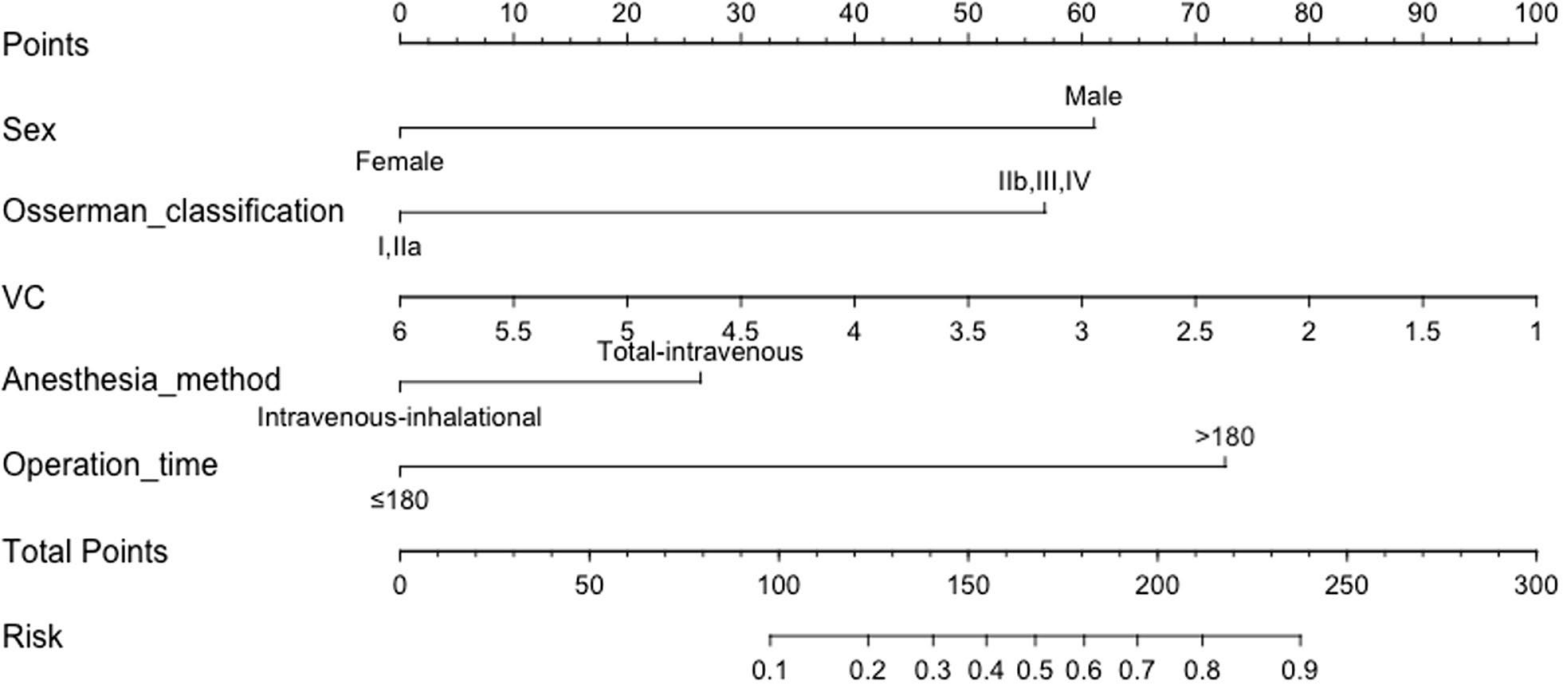
A nomogram predicting prolong ventilation for TAMG patients after thymectomy. The value of each of variable was given a score on the point scale axis. A total score could be easily calculated by adding each single score and locate it on the total points axis. Draw a line straight down to find the probability of post-operation ventilation

**Fig. 2:**
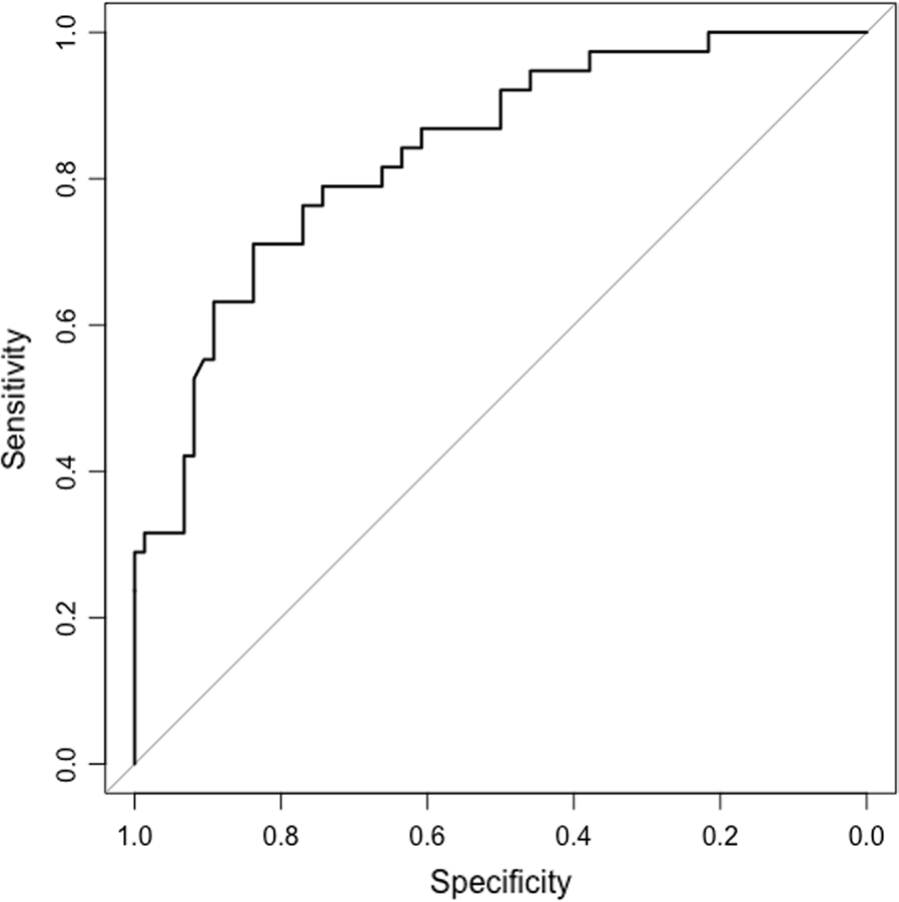
Receiver operating characteristic (ROC) curves of the nomogram in the training dataset. Area under the curve (AUC) is 0.835

**Fig. 3:**
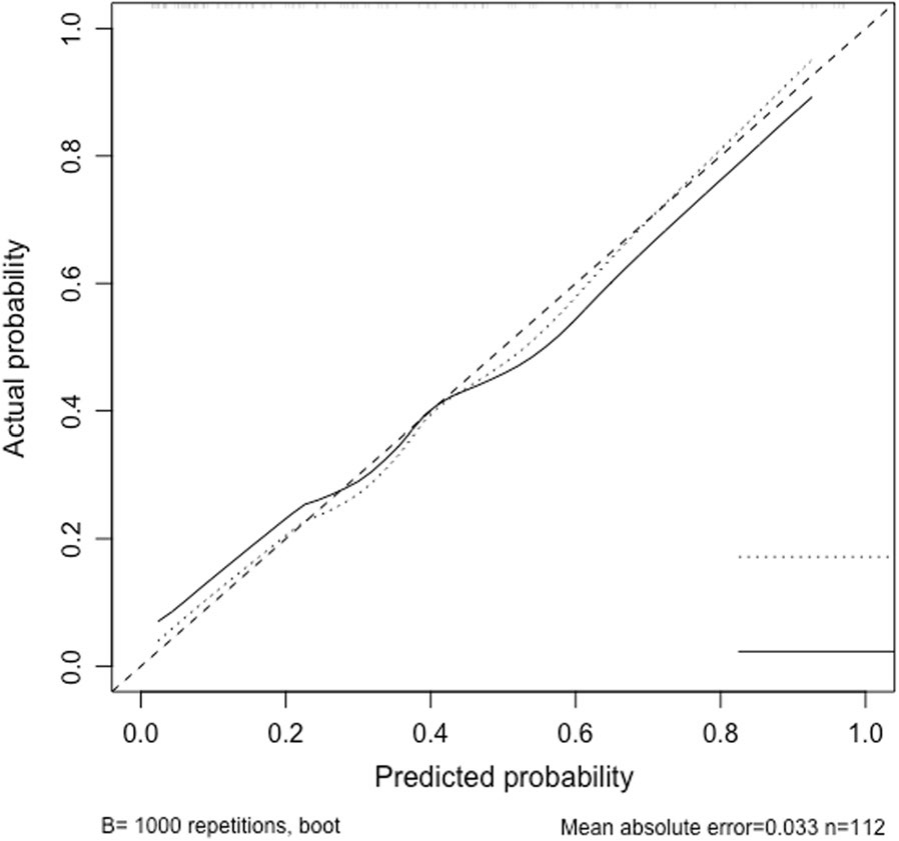
Calibration curve of the nomogram in the training set. The x-axis is the nomogram-predicted probability of postoperative ventilation and the y-axis is the actual rate

A nomogram is constructed based on the results of the multivariable logistic regression (Fig. 1). To evaluate the discrimination of the model and to reduce overfitting bias, internal validation was performed using a bootstrapping technique with 1000 resamples as qualified by C-index, which is more accurate than dividing the data into two parts when the dataset is small. In our study, the discrimination accuracy of the model is 0.835 (95% CI, 0.757–0.913) (Fig. 2), indicating excellent prediction accuracy of prolonged ventilation for TAMG patients after thymectomy. And the C-index for prediction was 0.835, and the calibration plot showed good agreement between the predicted and observed rates (*p* = 0.921, Hosmer–Lemeshow test, Fig. 3).

## Discussion

The weaning management of MG patients is a callange. Till now clinical judgment of whether extubation can be done after the operation is mostly based on clinical experience, which will result in premature or delayed extubation and increase the incidence of adverse events. So, grasping opportunity for weaning in time is meaningful. However, the need for prolonged ventilation is dependent on many factors. In this study, we integrated multiple clinical variables both the baseline clinical and the anesthesia, surgery factors to develop a logistic regression-based model to predict the need for prolonged ventilation for TAMG patients after thymectomy. We found that sex, VC, the Osserman classification of MG, the anesthesia method, and the duration of the operation were independent predictors. Not only this, but we also incorporate them into a user-friendly nomogram for predicting. Importantly, our nomogram had excellent discrimination properties, with an AUC of 83.5%.

In this study, patients were 54 ± 12.2 years old, and female was about 56.3%. Similar to the previous studies we find that prolonged postoperative ventilation is common in patients with MG patients, about 33.9% (38/112). Nearly one-third of patients (38/112, 33.9%) required postoperative ventilation, and the proportion of male patients was relatively high (60.5 vs. 36.5, *p* = 0.015). Furthermore, the average length of ventilation is 36.8 h. 7 patients need ventialtion for more than 7 days and 3 of them over 2 weeks. Besides, those patients who required prolonged ventilation had much longer hospital stays (Z =  − 5.490, *p* < 0.001).

As surgery is an inestimable challenge for MG patients, we not only focus on the preoperative factors but also analyse the possible related details of anesthesia and surgery, to explore the risk factors of postoperative ventilation. Surprisingly, the risk factors previously identified by Leventhal et al. [7] (history of pulmonary disease, duration of disease > 6 years, pyridostigmine dosage > 750 mg/day and VC < 2.9 L) were not completely consistent with our study. It may be attributed to several reasons. First, compared to the 12.5% (3/24) patients in Leventhal who had underlying chronic respiratory disease, only 8.0% (9/112) in our study. And 3 of them were tuberculosis and 2 for asthmas, emphysema and pneumonia. Additionally, we analyzed the actual pulmonary function of the patients by the pulmonary function tests before surgery and shown ventilatory reserve, maximum expiratory flow at 50% (MEF 50%), MEF 75% and MEF 25–75% are lower 89.80 (86.40, 92.90) %, but without significant destroy in both ventilation and diffusion function. But VC% were lower in prolonging ventilation group (*p* < 0.05). Besides, Multivariate analyses shown that VC was an independent risk factor of the need for postoperative ventilation. Second, some researchers found that the longer the preoperative course of MG, the more irreversible damage to the neuromuscular junction. And the average duration of the MG in our study was 60 (30, 80) days in our study. 63 patients with mild clinical symptoms, 49 patients were more serious with bulbar symptoms (Osserman Classification IIb). Compared with group 1, patients in group 2 have a longer duration of MG and with more severe symptoms (*p* = 0.001). Third, as the better knowledge of the pathology and physiology of TAMG, medical therapy strategy was more actively and standardized base on the latest clinical guideline. In our study, 55 (49.1%) patients received preoperative medicine, moreover, 12 of them use glucocorticoid in combination, 4 patients need a high dose of intravenous immune globulin and or plasma exchange and none of our patients suffered MC before surgery. But few patients were used medicine in combination therapy in previous studies. So, the value of 750 mg/day suggested by Leventhal et al. the average dose of preoperative pyridostigmine dose is much higher than 75 (0.180) mg/day in our study. Overall, the earlier diagnosis and the more effective management by use immunosuppressive in combination [14] make it easier to keep the patients in a stable state and a better outcome even after surgery.

Additionally, we thought the anesthesia and surgery could make big difference in the prognosis of patients. The univariable analysis shown total intravenous anesthesia, OT, long operation time, much blood loss, and having pulmonary wedge resection or pericardiotomy was associate with a higher risk of postoperative ventilation.

The open median sternotomyis the standard approach for thymectomy as its better visualization of the anatomical structures. However, VATS, the minimally invasive procedure, is considered for clinical stage I–II [18]. In our study, 89.3% of our patients are in clinical stage II, and all have undergone extended thymectomy. VATS is used in 96 (85.7%) patients, but significantly less in the prolonged ventilation group (*p* = 0.001). Besides, group 2 has a longer operation time and more blood loss (*P* < 0.001). We thought that surgery by VATS only follows the muscle fibers, with less damage to respiratory muscles, shooter incisions, and slighter postoperative pain, which maximal protecting the respiratory muscles by minimizing the restriction of the chest wall muscles movement and the impair of the respiratory driving force. And it has been proved that the reduction of postoperative incision is conducive to the recovery of lung function in the early postoperative period, which has a certain relationship with the protection of respiratory function [4], especially in the force of early inhalation and forceful expiration [5]. In short, as the previous studies have shown VATS has an equal if not superior oncological efficacy, better perioperative complications and survival outcomes [19, 20].

Although sedatives and volatile anesthetics can exacerbate MG, the best general anesthesia methods of MG patients in surgery have been contradictory. MG patients are more sensitive than normal to the neuromuscular depressant effects of halothane and isoflurane [21, 22]. Maddali et al. [23] reported the safety use of sevoflurane and propofol when real-time monitoring the depth of anesthesia in a small cohort of patients undergoing transthymectomy. In contrast, Nitahara et al. [24] have reported that sevoflurane inhibits neuromuscular transmission in a dose-dependent fashion. In our study, 60.7% of patients used intravenous-inhalational anesthesia by sevoflurane and propofol. And patients with total intravenous anesthesia have a lower risk of postoperative ventilation. Nondepolarizing NMBAs were used in 96.4% of patients, but there is no difference between two groups (*p* = 1.00). The complications of residual neuromuscular blockade of nodepolarizing NMBAs is common in MG patients. So, it is suggested that the amount given should be reduced to one-third or less, depending on the severity of MG disease. Regrettably, the type of nondepolarizing NMBAs we use varies among patients, so is incomparable between the drugs, and analysis their action, side effect in different anesthesia methods. Furthermore, we found more people received muscle relaxant antagonists in group 1 (*p* = 0.001). In general, the use of objective quantitative neuromuscular monitors is necessary when the NMBAs are used in MG patients, and the amount must be titrated to the individual.

## Limitation

There are several limitations of this study. First, the nature of retrospective and single center study had resulted in limited power of funding out all potential confounding factors. Second, our research analyzed the data of TAMG patients over 7 years as the TAMG is not a common disease, which might lead to significant heterogeneity among the study population. Nevertheless, the main management of MG patients doesn’t change during the study period in our medical center. Third, we don’t have a protocol on anesthesia, the method of general and regional anesthesia, the use of muscle relaxants is all dependent on the favorite of the anesthetist. So, additional prospective, large-scale, randomized and more methodologically rigorous studies are needed. In addition, although bootstrapping technology was used as an internal validation in our study, external validation is also needed.

## Conclusions

Various factors have been suggested to have influence on extubation after thymectomy for myasthenia patients. Among these, our study suggested that the sex, VC, Osserman classification, anesthesia method, operation time may influence the extubation for MG patients after thymectomy. And we also developed and validated a nomogram, hoping it will be useful for the clinical decision.

## Supplementary Information

**Additional file 1: Fig. S1.** Patient flow chart

**Additional file 2: Supplementary Table A.** Demographic, preoperative and pathological data

**Additional file 3: Supplementary Table B.** Univariable analyses for prolong ventilation for TAMG patients after thymectomy

## Data Availability

The authors declare that all data supporting the findings of this study are available within the article.

## Abbreviations

TAMG: Thymoma-associated myasthenia gravis
VC: Vital capacity
AUC: Area under the receiver operating characteristic curve
MG: Myasthenia gravis
MC: Myasthenic crisis
CT: Computed tomography
NMBAs: Neuromuscular blockers agents
HR: Heart rate
SBT: Spontaneous breathing test
PaO2: Arterial oxygen pressure
PaCO2: Arterial carbon dioxide pressure
RR: Respiratory rate
BMI: Body mass index
WHO: World Health Organization
MEF: Maximum expiratory flow
VIF: Variance inflation factor
