# Supplementary figures and images for: Risk factors of prolonged ventilation after thymectomy in thymoma myasthenia gravis patients
Source: J Cardiothorac Surg. 2021 Sep 27;16:275. doi: 10.1186/s13019-021-01668-8 (PMC8475491; doi:10.1186/s13019-021-01668-8)

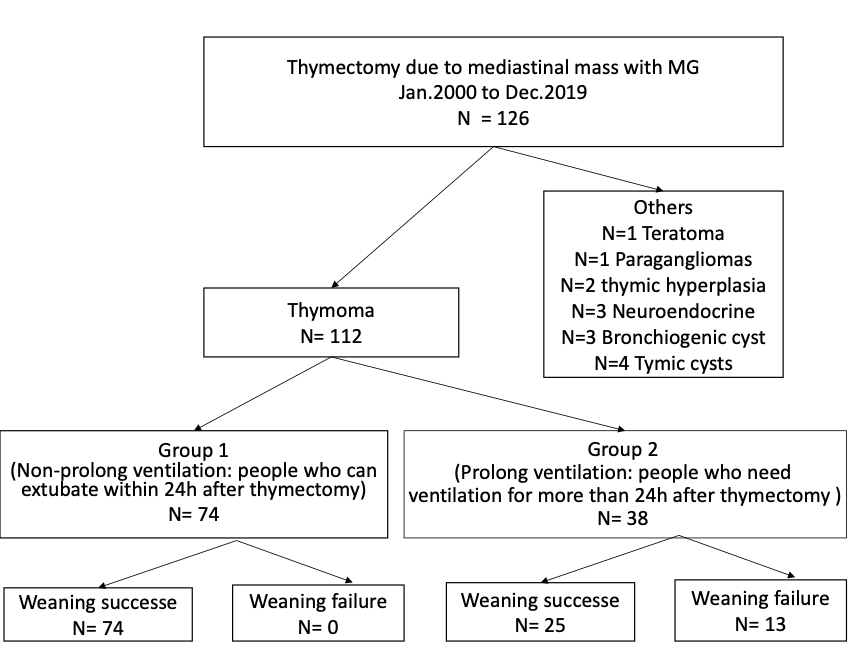

Supplement: Supplementary file 1 — Additional file 1: Fig. S1. Patient flow chart [file 13019_2021_1668_MOESM1_ESM.png]
