# Supplementary material for: Risk factors of prolonged ventilation after thymectomy in thymoma myasthenia gravis patients
Source: J Cardiothorac Surg. 2021 Sep 27;16:275. doi: 10.1186/s13019-021-01668-8 (PMC8475491; doi:10.1186/s13019-021-01668-8)
Supplement: Supplementary file 2 — Additional file 2: Supplementary Table A. Demographic, preoperative and pathological data [file 13019_2021_1668_MOESM2_ESM.docx]

| **Supplemental Table A.** **Demographic，preoperative and pathological data** | |
| --- | --- |
| Variable |  |
| Age (years), *M (Q_25_, Q_75_)* | 54.0±12.2  51.0(44.0,61.0) |
| Sex, male/female | 50/62 |
| BMI, *M (Q_25_, Q_75_)* | 24.2±4.0  24.2 (21.1,26.7) |
| Duration of the disease(day) | 60(30,80) |
| Diameter (cm), *M (Q_25_, Q_75_)* | 4.5(3.0,6.0) |
| Osserman classiﬁcation, n (%) |  |
| I | 34(30.4) |
| Iia | 29(25.9) |
| Iib | 37(33) |
| III | 10(8.9) |
| IV | 2(1.8)s |
| Preoperative dose of pyridostigmine | 75(0,180) |

BMI, Body mass index; MNT, Micronodular thymoma with lymphoid.
