# Supplementary material for: Risk factors of prolonged ventilation after thymectomy in thymoma myasthenia gravis patients
Source: J Cardiothorac Surg. 2021 Sep 27;16:275. doi: 10.1186/s13019-021-01668-8 (PMC8475491; doi:10.1186/s13019-021-01668-8)
Supplement: Supplementary file 3 — Additional file 3: Supplementary Table B. Univariable analyses for prolong ventilation for TAMG patients after thymectomy [file 13019_2021_1668_MOESM3_ESM.docx]

**Supplemental Table B. Univariable analyses for prolong ventilation after thymectomy for TAMG patients**

| Variables | VIF | OR | 95%CI | P-value |
| --- | --- | --- | --- | --- |
| Age(year) | 1.257 | 1.013 | 0.965, 1.064 | 0.590 |
| Gender(Male) | 2.032 | 0.187 | 0.040, 0.726 | 0.021 |
| BMI | 1.447 | 1.011 | 0.874, 1.172 | 0.884 |
| Complications | 1.204 | 0.852 | 0.264, 2.711 | 0.786 |
| Smoking | 1.665 | 2.081 | 0.457, 9.894 | 0.344 |
| VC(L) | 1.883 | 0.609 | 0.271, 1.260 | 0.200 |
| Osserman classiﬁcation (IIb-IV) | 1.672 | 7.193 | 2.112, 29.197 | 0.002 |
| Duration of the disease(day) | 1.289 | 0.999 | 0.998,1.001 | 0.535 |
| Preoperative dose of pyridostigmine (mg) | 1.382 | 1.000 | 0.995, 1.006 | 0.880 |
| Size of thymomas (cm) | 2.074 | 0.985 | 0.773, 1.256 | 0.904 |
| Anesthesia method (Total-intravenous) | 1.423 | 2.357 | 0.716, 8.462 | 0.168 |
| Nondepolarizing NMBAs | 1.468 | 1.856 | 0.237, 19.433 | 0.566 |
| Vasoactive agents | 1.534 | 0.412 | 0.101, 1.433 | 0.183 |
| Surgical procedure (OT) | 2.244 | 0.616 | 0.177, 4.625 | 0.238 |
| Phrenic never or diaphragm injury | 1.704 | 0.896 | 0.215, 3.490 | 0.875 |
| Plumonary wedge resection | 1.590 | 2.056 | 0.412, 10.729 | 0.379 |
| Pericardiotomy | 1.698 | 2.399 | 0.599, 10.207 | 0.219 |
| Operative blood loss(ml) | 1.282 | 0.989 | 0.899, 1.001 | 0.255 |
| Operation time >180min | 2.120 | 11.494 | 2.615, 62.321 | 0.002 |

VC, Vital capacity; NMBAs, Neuromuscular blockers agents; OT, Open transsternal;
